# New Drugs, Appliances, and Things Medical

**Published:** 1892-12-10

**Authors:** 


### NEW DRUGS, APPLIANCES, AND THINGS MEDICAL.

### RESTRAINT OR REMOVAL JACKET FOR INSANE PATIENTS.

This jacket is effective and comfortable, but is too elaborate and unnecessarily formidable in appearance. Though restraint should certainly be used when it is required, still the garment employed should suggest the idea as little as possible. The jacket would be more easily put on if made to fasten behind, and such a harness-like arrangement of straps and buckles might be avoided, and, indeed, is not needed. The method of disposing of the arms is good. It would be a useful article for relieving officers who have to remove an excited patient to the asylum. It would give the patient the minimum of discomfort, and beneath a mackintosh would be quite unnoticed. The jackets are to be procured from Messrs. Pocock Brothers, 235, Southwark Bridge Road. The price complete with locked buckle and straps, is 35s.

